# Current state and future perspectives of spinal navigation and robotics—an AO spine survey

**DOI:** 10.1016/j.bas.2024.104165

**Published:** 2024-12-18

**Authors:** Stefan Motov, Vicki M. Butenschoen, Philipp E. Krauss, Anand Veeravagu, Kelly H. Yoo, Felix C. Stengel, Nader Hejrati, Martin N. Stienen

**Affiliations:** a Spine Center of Eastern Switzerland, Kantonsspital St. Gallen & Medical School of St. Gallen, St.Gallen, Switzerland; b Department of Neurosurgery, Kantonsspital St. Gallen & Medical School of St. Gallen, St.Gallen, Switzerland; c Department of Neurosurgery, Klinikum Rechts der Isar, Technical University of Munich, Munich, Germany; d Department of Neurosurgery, University Hospital Augsburg, Augsburg, Germany; e Neurosurgery Artificial Intelligence Lab, Stanford University School of Medicine, Stanford, CA, USA; f Department of Neurosurgery, Stanford University School of Medicine, Stanford, CA, USA

**Keywords:** Spine surgery, Instrumentation, Navigation, Robotics, Survey, Utilization

## Abstract

**Introduction:**

The use of robotics in spine surgery has gained popularity. This study aims to assess the current state of robotics and raise awareness of its educational implications.

**Research question:**

What are the current adoption trends and barriers to the implementation of robotic assistance in spine surgery?

**Material and methods:**

An online questionnaire comprising 27 questions was distributed to AO spine members between October 25th and November 13th, 2023, using the SurveyMonkey platform (https://www.surveymonkey.com; SurveyMonkey Inc., San Mateo, CA, USA). Statistical analyses (descriptive statistics, Pearson Chi-Square tests) and generation of all graphs were performed using SPSS Version 29.0.1.0 (IBM SPSS Statistic).

**Results:**

We received 424 responses from AO Spine members (response rate = 9.9 %). The participants were mostly board-certified orthopedic surgeons (46 %, n = 195) and neurosurgeons (32%, n = 136). While 49% (n = 208) of the participants reported occasional or frequent use of navigation assistance, only 18 % (n = 70) indicated the use of robotic assistance for spinal instrumentation. A significant difference based on the country's median income status (p < 0.001) and the respondent's number of annual instrumentation procedures (p < 0.001) has been observed. While 11 % (n = 47) of all surgeons use a spinal robot frequently, 36 % (n = 153) of the participants stated they don't need a robot from a current perspective. Most participants (77%, n = 301) concluded that high acquisition costs are the primary barrier for the implementation of robotics.

**Discussion and conclusion:**

Although the hype for robotics in spine surgery increased recently, robotic systems remain non-standard equipment due to cost constraints and limited usability.

## Highlights

• Educational Implications of robotics.
• Low utilization in spine surgery.
• High acquisition costs.

## 1 Introduction

Robotics has revolutionized spine surgery since the introduction of the first spine robot, the SpineAssist (Mazor Robotics Ltd., Caesarea, Israel), in 2004(1). Since then, the indications and their implementation expanded(D'Souza et al., 2019; Stumpo et al., 2021). Two of the greatest impacts of robotic guidance systems have been their use in minimally invasive surgical (MIS) procedures and spine surgery training(Liounakos et al., 2021). Planning and execution tools enabled a broader use of MIS approaches (Patel et al., 2023) and better management of challenging patient anatomy, even in complex revision (Haider et al., 2022) and deformity cases (Haider et al., 2023). Despite these advancements, skepticism persists among spine surgeons regarding the true benefits of spinal robotics compared to real-time image-guided navigation, largely due to high acquisition costs(Kochanski et al., 2019) and perceived limited versatility.

While a relevant number of spine centers have acquired a spinal robot, it is unclear how often the robotic systems are utilized and for which indications they are considered beneficial. Even if cost-effectiveness has been described as superior in certain settings(Menger et al., 2018), this likely does not apply on a broad scale and an international basis. In response to this need, we designed a survey aimed at providing a landscape assessment of current practices and creating educational awareness for spinal robotics throughout the AO regions. This survey seeks to address existing gaps in knowledge and provide valuable insights into the utilization and perceived benefits of spinal robotics in clinical practice.

## 2 Methods

### 2.1 Survey administration

A web-based survey was conducted among AO spine members between October 25th and November 13th^,^ 2023. The 27-multiple choice questionnaire, hosted on the SurveyMonkey platform (https://www.surveymonkey.com; SurveyMonkey Inc., San Mateo, CA, USA), aimed to assess the utilization of navigation and spinal robotics in current practice. The survey was created using and distributed by the AO Spine email platform, with a reminder sent on November 8th. Additionally, promotion via social networks (Facebook, Instagram, LinkedIn) was utilized to enhance awareness and participation.

### 2.2 Survey design

The questionnaire predominantly comprised multiple-choice questions, of which the first five included data on the demographic, professional, and geographic background of the participants as well as general characteristics. Subsequent questions (Haider et al., 2023; Kochanski et al., 2019; Menger et al., 2018; Bratschitsch et al., 2019; Tjardes et al., 2010; Meng et al., 2016) explored the level of participants' expertise, departmental practices regarding instrumentation/fusion procedures, and the use of a spinal navigation system. Several questions (Soliman et al., 2023; Lange et al., 2021; Tarawneh et al., 2021) centered around the participants’ experience with spinal robotics. Those who stated to use a robotic system occasionally (1–2 times/a month) or frequently (1–2 times a week) were asked to answer questions (Baldwin Kdk et al., 2022; De Vega et al., 2022; Matur et al., 2023; Li et al., 2020) addressing the benefits of spinal robotics in their practice. The last part of the survey (questions 19–27) contained questions on expectations, future innovations, and economic aspects of spinal robotics. The complete survey is included in Appendix I.

### 2.3 Statistical analysis

All statistical analyses and generation of all graphs were performed using SPSS Version 29.0.1.0 (IBM SPSS Statistic). Descriptive statistics were employed, describing the responses as count (percent) and mean (standard deviation; SD). Graphical illustrations of results were used to explore relationships. We used Pearson-Chi-Square tests to analyze the influence of co-variables on dichotomous questions. Workplaces based on academic level, level of expertise based on the number of instrumentation procedures, geographic backgrounds based on the country's median income status measured by the World Bank Country Classification, and profession have been dichotomized. Results were considered significant at p-values <0.05.

## 3 Results

### 3.1 Survey responses

The estimated number of AO Spine members invited to respond to the survey via initial email blast was 4.300. Of these, we received 424 responses (response rate = 9.9 %). No datasets were removed due to duplication.

### 3.2 Demographics and characteristics of participants

The mean age of participants was 43 ± 10 years, with a majority being male (89%, n = 379) in the age group of 36–45 (n = 171; 40.3%). Most participants identified as board-certified orthopedic surgeons (46%, n = 195) or board-certified neurosurgeons (32%, n = 136), primarily from Europe (37%, n = 155) or Asia/Middle East (30%, n = 128). Most respondents originated from High Income Countries (HIC; n = 233; 54.6%). There was an equal distribution of participants from academic (n = 212; 50%) and non-academic (n = 212; 50%) institutions. The level of experience of participants varied; 24% (n = 103) indicated a low rate (n = 0–30), 48% (n = 205) a moderate rate (n = 31–120), and 28% (n = 116) a high rate (>121) of annual instrumentation procedures per capita. Further demographic characteristics are included in Table 1.

**Table 1:** Patient demographics data including professional background, workplace, region of practice, participants’ age, and number of annual instrumentations.

	Overall (n = 424; 100%)
**Professional background** Board-certified neurosurgeon Board-certified orthopedic surgeon Board-certified trauma surgeon Residents Other	n = 136 (31.9%) n = 195 (45.7%) n = 11 (2.6%) n = 55 (12.9%) n = 27 (6.3%)
**Workplace**	
University hospital Public, non-university hospital/practice Private hospital/practice Other	n = 210 (49.5%) n = 78 (18.4%) n = 121 (28.5%) n = 15 (3.5%)
**Academic Workplace** Academic Non-Academic	212 (49.6%) 212 (49.6%)
**Region of practice**	
Europe Asia & Middle East Africa North America South America Australia & New Zealand **Region of practice based on World Bank Country Classification** Low & Middle Income Country (LMIC) High Income Country (HIC) **Number of annual instrumentations**	n = 155 (36.6%) n = 128 (30.2%) n = 24 (5.7%) n = 65 (15.3%) n = 49 (11.6%) n = 3 (0.7%) 191 (44.7%) 233 (54.6%)
Low (0–30) Middle (31–120) High (>120)	n = 103 (24.3%) n = 207 (48.8%) n = 114 (26.9%)
**Age categories** 25–35 36–45 46–55 56–65 65+	n = 102 (24.1%) n = 171 (40.3%) n = 96 (22.6%) n = 40 (9.4%) n = 15 (3.5%)

### 3.3 Use of navigation assistance in spine surgery

The majority of participants reported using either free-hand (35%, n = 148) and/or free-hand fluoroscopy (57%, n = 243) techniques for pedicle screw insertion (participants were able to provide multiple answers to this question). A smaller percentage utilized navigation assistance (42%, n = 177), with even fewer employing robotic assistance (16%, n = 68).

**Fig. 1:**
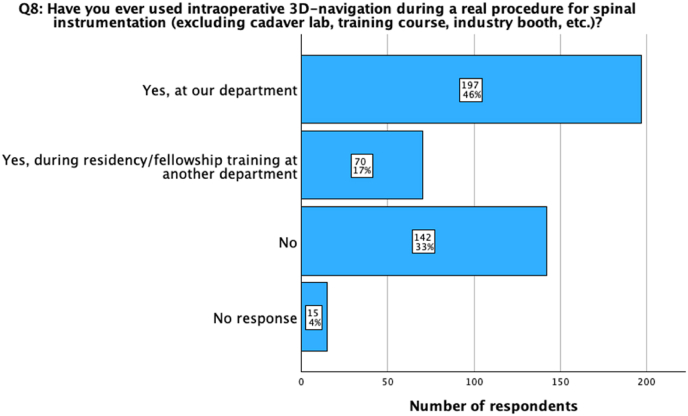
Question 8: Have you ever used intraoperative 3D-navigation during a real procedure for spinal instrumentation (excluding cadaver lab, training course, industry booth, etc.)? Almost half of the respondents (46%) indicated they have used spinal navigation at their department and further 17% during residency or fellowship at another department. One third (33%) of the participants stated that they have never used intraoperative 3D-navigation during a real patient procedure.

Thirty-four percent of participants (n = 142) reported never using intraoperative 3D navigation during real patient procedures (excluding cadaver lab, training course, industry booth, etc.) before, while 17% (n = 70) indicated only using it during residency/fellowship at another department (Fig. 1, Table 2). There was a significant difference in the prior use of navigation during a real patient procedure for spinal instrumentation based on the professional background, with board-certified neurosurgeons demonstrating higher familiarity (78% of neurosurgeons, n = 106) compared to orthopedic (64% of orthopedic surgeons, n = 124) and trauma surgeons (46% of trauma surgeons, n = 5; p < 0.001) (Table 2).

**Table 2:** Questions on navigation assistance in spine surgery stratified for country median income status, workplace, number of annual instrumentation procedures, and professional background of the participants. Income status, number of annual instrumentation procedures, and professional background significantly influence the use of spinal navigation (p < 0.01). Board-certified orthopedic surgeons and neurosurgeons from HIC with a middle or high number of annual instrumentation procedures are more likely to use spinal navigation for instrumentation procedures individually or in their departments. Significant results have been indicated through ∗, nearly significant through ∗∗.

	Total	Country median income status			Workplace			Number of annual instrumentation procedures			
**9) Does your department own/use a navigation system for spinal instrumentation?**	N = 424 (100%)	LMIC (n = 191; 45%)	HIC (n = 233; 55%)	p-value	Academic (n = 212; 50%)	Non-Academic (n = 212; 50%)	p-value	Low (n = 103; 24.3%)	Middle (n = 207; 48.8%)	High (n = 114; 26.9%)	p-value
No response	15 (3.5%)	10 (2.4%)	5 (1.2%)	p < 0.001∗	9 (2.1%)	6 (1.4%)	p = 0.298	8 (1.9%)	6 (1.4%)	1 (0.2%)	p < 0.001∗
Yes, and we use it frequently (1-2x/week)	138 (32.5%)	28 (6.6%)	110 (25.9%)		76 (17.9%)	62 (14.6%)		13 (3.1%)	78 (18.4%)	47 (11.1%)	
Yes, but we use it only occasionally (1-2x/month)	70 (16.5%)	18 (4.2%)	52 (12.3%)		34 (8%)	36 (8.5%)		12 (2.8%)	40 (9.4%)	18 (4.2%)	
Yes, but we don't use it at all currently	33 (7.8%)	19 (4.5%)	14 (3.3%)		12 (2.8%)	21 (5%)		8 (1.9%)	16 (3.8%)	9 (2.1%)	
No but we are planning to acquire one	113 (26.7%)	81 (19.1%)	32 (7.5%)		58 (13.7%)	55 (13%)		33 (7.8%)	52 (12.3%)	28 (6.6%)	
No, and we don't want/need it from a current perspective	55 (13%)	35 (8.3%)	20 (4.7%)		23 (5.4%)	32 (7.5%)		29 (6.8%)	15 (3.5%)	11 (2.6%)	
**11) Do you personally use a navigation system for spinal instrumentation in your surgical routine?**	N = 424 (100%)	LMIC (n = 191; 45%)	HIC (n = 233; 55%)	p < 0.001∗	Academic (n = 212; 50%)	Non-Academic (n = 212; 50%)	p = 0.250	Low (n = 103; 24.3%)	Middle (n = 207; 48.8%)	High (n = 114; 26.9%)	p < 0.001∗
Other, please specify	23 (5.4%)	13 (3.1%)	10 (2.4%)		15 (3.5%)	8 (1.9%)		11 (2.6%)	9 (2.1%)	3 (0.7%)	
Yes, routinely (standard for spinal instrumentation)	96 (22.6 %)	16 (3.8%)	80 (18.9 %)		54 (12.7%)	42 (9.9%)		11 (2.6%)	52 (12.3%)	33 (7.8%)	
Yes, but only in selected cases (not for standard cases)	99 (23.3 %)	32 (7.5%)	67 (15.8%)		46 (10.8%)	53 (12.5%)		10 (2.4%)	60 (14.2%)	29 (6.8%)	
No, but I would like to	170 (40.1%)	120 (28.3%)	50 (11.8%)		78 (18.4%)	92 (21.7%)		64 (15.1%)	70 (16.5%)	36 (8.5%)	
No, and I don't want to	36 (8.5%)	10 (2.4%)	26 (6.1%)		19 (4.5%)	17 (4%)		7 (1.7%)	16 (3.8%)	13 (3.1%)	
**8) Have you ever used intraoperative 3D-navigation during a real patient procedure for spinal instrumentation?**	N = 424 (100%)	LMIC (n = 191; 45%)	HIC (n = 233; 55%)	p < 0.001∗	Academic (n = 212; 50%)	Non-Academic (n = 212; 50%)	p = 0.758	Low (n = 103; 24.3%)	Middle (n = 207; 48.8%)	High (n = 114; 26.9%)	p < 0.001∗
No response	15 (3.5%)	10 (2.4%)	5 (1.2%)		9 (2.1%)	6 (1.4%)		8 (1.9%)	6 (1.4%)	1 (0.2%)	
No	142 (33.5%)	95 (22.4%)	47 (11.1%)		67 (15.8%)	75 (17.7%)		61 (14.4%)	57 (13.4%)	24 (5.7%)	
Yes, during residency/fellowship training at another department	70 (16.5%)	35 (8.3%)	35 (8.3%)		35 (8.3%)	35 (8.3%)		13 (3.1%)	36 (8.5%)	21 (5%)	
Yes, at our department	197 (46.5%)	51 (12%)	146 (34.4%)		101 (23.8%)	96 (22.6%)		21 (5%)	108 (25.5%)	68 (16%)	

	Total	Professional background					
**9) Does your department own/use a navigation system for spinal instrumentation?**	N = 424 (100%)	Board-certified neurosurgeon (n = 136; n = 32.1%)	Board-certified orthopedic surgeon (n = 195; 46%)	Board-certified trauma surgeon (n = 11; 2.6%)	Resident (n = 55; 13%)	Other (n = 27; 6.4%)	p-value
No response	15 (3.5%)	2 (0.5%)	5 (1.2%)	1 (0.2%)	4 (0.9%)	3 (0.7%)	p = 0.012∗∗
Yes, and we use it frequently (1-2x/week)	138 (32.5%)	59 (13.9%)	54 (12.7%)	3 (0.7%)	19 (4.5%)	3 (0.7%)	
Yes, but we use it only occasionally (1-2x/month)	70 (16.5%)	23 (5.4%)	33 (7.8%)	1 (0.2%)	0	1 (0.2%)	
Yes, but we don't use it at all currently	33 (7.8%)	13 (3.1%)	18 (4.2%)	1 (0.2%)	0	1 (0.2%)	
No but we are planning to acquire one	113 (26.7%)	30 (7.1%)	56 (13.2%)	3 (0.7%)	15 (3.5%)	9 (2.1%)	
No, and we don't want/need it from a current perspective	55 (13%)	9 (2.1%)	29 (6.8%)	2 (0.5%)	8 (1.9%)	7 (1.7%)	
**11) Do you personally use a navigation system for spinal instrumentation in your surgical routine?**	N = 424 (100%)	Board-certified neurosurgeon (n = 136; 32.1%)	Board-certified orthopedic surgeon (n = 195; 46%)	Board-certified trauma surgeon (n = 11; 2.6%)	Resident (n = 55; 13%)	Other (n = 27; 6.4%)	p < 0.001∗
Other, please specify	23 (5.4%)	3 (0.7%)	9 (2.1%)	1 (0.2%)	5 (1.2%)	5 (1.2%)	
Yes, routinely (standard for spinal instrumentation)	96 (22.6%)	46 (10.8%)	36 (8.5%)	1 (0.2%)	12 (2.8%)	1 (0.2%)	
Yes, but only in selected cases (not for standard cases)	99 (23.3%)	38 (9%)	44 (10.4%)	3 (0.7%)	9 (2.1%)	5 (1.2%)	
No, but I would like to	170 (40.1%)	43 (10.1%)	81 (19.1%)	4 (0.9%)	28 (6.6%)	14 (3.3%)	
No, and I don't want to	36 (8.5%)	6 (1.4%)	25 (5.9%)	2 (0.5%)	1(0.2%)	2 (0.5%)	
**8) Have you ever used intraoperative 3D-navigation during a real patient procedure for spinal instrumentation?**	N = 424 (100%)	Board-certified neurosurgeon (n = 136; 32.1%)	Board-certified orthopedic surgeon (n = 195; 46%)	Board-certified trauma surgeon (n = 11; 2.6%)	Resident (n = 55; 13%)	Other (n = 27; 6.4%)	p < 0.001∗
No response	15 (3.5%)	2 (0.5%)	5 (1.2 %)	1 (0.2%)	4 (0.9%)	3 (0.7%)	
No	142 (33.5%)	28 (6.6%)	66 (15.6%)	5 (1.2%)	26 (6.1%)	17 (4%)	
Yes, during residency/fellowship training at another department	70 (16.5%)	22 (5.2%)	41 (9.7%)	0	5 (1.2 %)	2 (0.5%)	
Yes, at our department	197 (46.5%)	84 (19.8%)	83 (19.6%)	5 (1.2%)	20 (4.7%)	5 (1.2%)	

Nearly half of the participants (49%, n = 208) expressed to use a navigation system for spinal instrumentation at their institution occasionally (1–2 times/month; 16.5%, n = 70) or frequently (1–2 times/week; 32.5%, n = 138). Thirteen percent (n = 55) stated they don't want to acquire a navigation system at all. There was again a significant difference based on the subspecialty regarding the utilization of a spinal navigation system; a significantly higher percentage of board-certified neurosurgeons (60% of neurosurgeons, n = 82) indicated using a spinal navigation system frequently or occasionally, compared to board-certified orthopedic surgeons (45% of orthopedic surgeons, n = 87; p < 0.001). Nearly half of the participants (49.5%; n = 210) indicated that the spinal navigation system at their institution included intraoperative 3D imaging: a 3D C-Arm (16%; n = 68), cone beam-CT (e.g., O-Arm; 24.5%; n = 104), or CT (e.g., Airo-CT; 9%; n = 38).

**Fig. 2:**
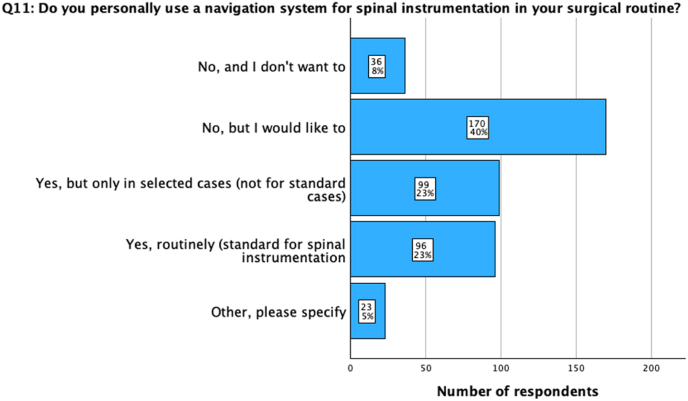
Question 11: Do you personally use a navigation system for spinal instrumentation in your surgical routine? 46 % of the surgeons either use spinal navigation routinely or in selected cases in their individual routine for spinal instrumentation. While additional 40 % would like to use one, 8 % would not want to in their practice.

Most participants (86%, n = 365) reported either routinely using (23%, n = 96), occasionally using in selected cases (23%, n = 99), or expressing a desire to use (40%, n = 170; Fig. 2) a navigation system for spinal instrumentation in their surgical routine. Subgroup analysis based on the level of surgical expertise demonstrated a lower threshold for routine or occasional utilization of spinal navigation among surgeons with high (54%, n = 62) or moderate (54%, n = 112) rates of instrumentation procedures compared to surgeons with low rates of instrumentations per year (21%, n = 21; p < 0.001). Based on geographic disparities, significantly more participants from HIC (70%; n = 162) than from LMIC (24%; n = 46) indicated using spinal navigation routinely or occasionally in selected cases (p < 0.001). Based on academic vs non-academic background, there was no statistical difference regarding the utilization of spinal navigation between the departments (p = 0.298).

### 3.4 Current use of robotics in spine surgery

**Fig. 3:**
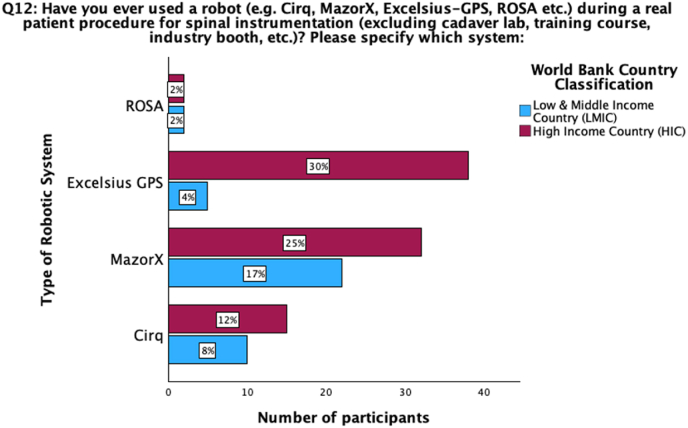
Question 12: Have you ever used a robot (e.g. Cirq, MazorX, Excelsius-GPS, ROSA etc.) during a real patient procedure for spinal instrumentation (excluding cadaver lab, training course, industry booth, etc.)? The figure depicts the most commonly used robotic systems based on the participants' region of practice depending on income status. Excelsius GPS is the most commonly used system in HIC, Mazor X is the most commonly used robot in LMIC.

**Fig. 4:**
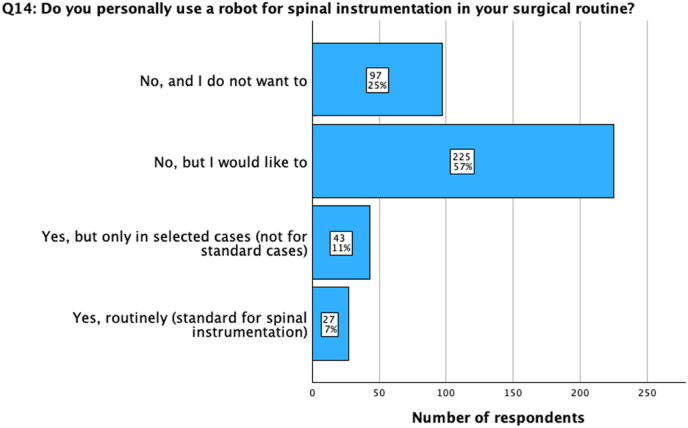
Question 14: Do you personally use a robot for spinal instrumentation in your surgical routine? Compared to spinal navigation, participants are less prone to use a robot for spinal instrumentation procedures (25%). Still, only a low number of surgeons apply robotics on a routine basis for spinal instrumentation (7%), while more surgeons (11%) use it in selected cases.

Overall, 21% (n = 89) of participants stated using a robot during a real patient procedure for spinal instrumentation at their department, with an additional 12% (n = 52) utilizing spinal robotics during residency or fellowship training at another department (Table 3). 63% (n = 267) of all participants indicated not having used a spinal robot ever before. The most frequently used robotic systems were MazorX (Medtronic; n = 54, 43%), Excelsius GPS (Globus Medical; n = 43, 34%) and Cirq (Brainlab; n = 25, 20%). Based on country's income status, it appeared that Excelsius GPS (n = 38; 30%) was the most commonly used robotic system in HIC and MazorX in LMIC (n = 22; 17%) (Fig. 3, Fig. 4).

**Table 3:** Questions on robotics assistance in spine surgery stratified for country's median income status, workplace, number of annual instrumentation procedures, and professional background of the participants. The country's median income status and the number of annual instrumentation procedures significantly influence the responses regarding the use and possession of robotics in spine surgery (p < 0.01). Mostly respondents from HIC with a middle or high number of instrumentation procedures own/use a robotic system for spinal instrumentation. A higher number of board-certified neurosurgeons and orthopedic surgeons from primarily LMIC with a middle number of instrumentation procedures would be interested to acquire a robotic system. Significant results have been indicated through ∗, nearly significant through ∗∗.

	Total	Country median income status			Workplace			Number of annual instrumentation procedures			
**14) Do you personally use a robot for spinal instrumentation in your surgical routine?**	N = 392(98%)	LMIC (n = 178; 45.4%)	HIC (n = 214; 54.6%)	p-value	Academic (n = 193; 49.2%)	Non-Academic (n = 199; 50.8%)	p-value	Low (n = 91; 23.2%)	Middle (n = 193; 49.2%)	High (n = 108; 27.6%)	p-value
Yes, routinely (standard for spinal instrumentation	27 (6.9 %)	8 (2%)	19 (4.8%)	p < 0.001∗	12 (3.1%)	15 (3.8%)	p = 0.9	3 (0.8%)	9 (2.3%)	15 (3.8%)	p < 0.001∗
Yes, but only in selected cases (not for standard cases)	43 (11 %)	15 (3.8%)	28 (7.1%)		23 (5.9%)	20 (5.1%)		2 (0.5%)	23 (5.9%)	18 (4.6%)	
No, but I would like to	225 (57.4%)	128 (32.7%)	97 (24.7%)		111 (28.3%)	114 (29.1%)		71 (18.1%)	109 (27.8%)	45 (11.5%)	
No, and I do not want to	97 (24.7%)	27 (6.9%)	70 (17.9%)		47 (12%)	50 (12.8%)		15 (3.8%)	52 (13.3%)	30 (7.7%)	
**13) Does your department own/use a robot for spinal instrumentation?**	N = 424 (100%)	LMIC (n = 191; 45%)	HIC (n = 233; 55%)	p < 0.001∗	Academic (n = 212; 50%)	Non-Academic (n = 212; 50%)	p = 0.348	Low (n = 103; 24.3%)	Middle (n = 207; 48.8%)	High (n = 114; 26.9%)	p < 0.001∗
No response	17 (4%)	11 (2.6%)	6 (1.4%)		10 (2.4%)	7 (1.7%)		10 (2.4%)	6 (1.4%)	1 (0.2%)	
Yes, and we use it frequently (1-2x/week)	47 (11.1%)	12 (2.8%)	35 (8.3%)		20 (4.7%)	27 (6.4%)		3 (0.7%)	23 (5.4%)	21 (5%)	
Yes, but we use it only occasionally (1-2x/month)	34 (8%)	8 (1.9%)	26 (6.1%)		22 (5.2%)	12 (2.8%)		4 (0.9%)	16 (3.8%)	14 (3.3%)	
Yes but we don't use it at all currently	23 (5.4%)	8 (1.9%)	15 (3.5%)		11 (2.6%)	12 (2.8%)		3 (0.7%)	12 (2.8%)	8 (1.9%)	
No, but we are planning to acquire one	150 (35.4%)	92 (21.7%)	58 (13.7%)		78 (18.4%)	72 (17%)		29 (6.8%)	82 (19.3%)	39 (9.2%)	
No and we don't want/need it from a current perspective	153 (36.1 %)	60 (14.2%)	93 (21.9%)		71 (16.7%)	82 (19.3%)		54 (12.7%)	68 (16%)	31 (7.3%)	
**12) Have you ever used a robot during a real patient procedure for spinal instrumentation?**	N = 424 (100%)	LMIC (n = 191; 45%)	HIC (n = 233; 55%)	p = 0.002∗	Academic (n = 212; 50%)	Non-Academic (n = 212; 50%)	p = 0.757	Low (n = 103; 24.3%)	Middle (n = 207; 48.8%)	High (n = 114; 26.9%)	p < 0.001∗
No response	16 (3.8%)	10 (2.4%)	6 (1.4%)		10 (2.4%)	6 (1.4%)		9 (2.1%)	6 (1.4%)	1 (0.2%)	
Yes, during residency/fellowship training at another department	52 (12.3%)	19 (4.5%)	33 (7.8%)		27 (6.4%)	25 (5.9%)		8 (1.9%)	32 (7.5%)	12 (2.8%)	
Yes, at our department	89 (21%)	24 (5.7%)	65 (15.3%)		44 (10.4%)	45 (10.6%)		6 (1.4%)	44 (10.4%)	39 (9.2%)	
No	267 (63%)	138 (32.5%)	129 (30.4%)		131 (30.9%)	136 (32.1%)		80 (18.9%)	125 (29.5%)	62 (14.6%)	

	Total	Professional background					
**14) Do you personally use a robot for spinal instrumentation in your surgical routine?**	N = 392 (98%)	Board-certified neurosurgeon (n = 131; 33.4%)	Board-certified orthopedic surgeon (n = 185; 47.2%)	Board-certified trauma surgeon (n = 6; 1.5 %)	Resident (n = 47; 12%)	Other (n = 23; 5.9%)	p-value
Yes, routinely (standard for spinal instrumentation	27 (6.9 %)	9 (2.3%)	15 (3.8%)	1 (0.3%)	1 (0.3%)	1 (0.3%)	p = 0.659
Yes, but only in selected cases (not for standard cases)	43 (11 %)	16 (4.1%)	20 (5.1%)	0 (0%)	6 (1.5%)	1 (0.3%)	
No, but I would like to	225 (57.4%)	80 (20.4%)	97 (24.7%)	3 (0.8%)	29 (7.4%)	16 (4.1%)	
No, and I do not want to	97 (24.7%)	26 (6.6%)	53 (13.5%)	2 (0.5%)	11 (2.8%)	5 (1.3%)	
**13) Does your department own/use a robot for spinal instrumentation?**	N = 424 (100%)	Board-certified neurosurgeon (n = 136; 32.1%)	Board-certified orthopedic surgeon (n = 195; 46%)	Board-certified trauma surgeon (n = 11; 2.6 %)	Resident (n = 55; 13%)	Other (n = 27; 6.4%)	p = 0.006∗
No response	17 (4%)	2 (0.5%)	5 (1.2%)	2 (0.5%)	5 (1.2%)	3 (0.7%)	
Yes, and we use it frequently (1-2x/week)	47 (11.1%)	15 (3.5%)	23 (5.4%)	2 (0.5%)	5 (1.2%)	2 (0.5%)	
Yes, but we use it only occasionally (1-2x/month)	34 (8%)	10 (2.4%)	16 (3.8%)	0 (0%)	8 (1.9%)	0 (0%)	
Yes but we don't use it at all currently	23 (5.4%)	13 (3.1%)	9 (2.1%)	0 (0%)	1 (0.2%)	0 (0%)	
No, but we are planning to acquire one	150 (35.4%)	53 (12.5%)	73 (17.2%)	4 (0.9%)	10 (2.4%)	10 (2.4%)	
No and we don't want/need it from a current perspective	153 (36.1 %)	43 (10.1%)	69 (16.3%)	3 (0.7%)	26 (6.1%)	12 (2.8%)	
**12) Have you ever used a robot during a real patient procedure for spinal instrumentation?**	N = 424 (100%)	Board-certified neurosurgeon (n = 136; 32.1%)	Board-certified orthopedic surgeon (n = 195; 46%)	Board-certified trauma surgeon (n = 11; 2.6 %)	Resident (n = 55; 13%)	Other (n = 27; 6.4%)	p = 0.122
No response	16 (3.8%)	2 (0.5%)	5 (1.2%)	1 (0.2%)	5 (1.2%)	3 (0.7%)	
Yes, during residency/fellowship training at another department	52 (12.3%)	21 (5%)	22 (5.2%)	1 (0.2%)	4 (0.9%)	4 (0.9%)	
Yes, at our department	89 (21%)	34 (8%)	41 (9.7%)	2 (0.5%)	9 (2.1%)	3 (0.7%)	
No	267 (63%)	79 (18.6%)	127 (30%)	7 (1.7%)	37 (8.7%)	17 (4%)

There was a significant difference in robotic usage in the departments based on geographic origin, with the majority of users coming from Europe, Asian & Middle East countries, or North America (p = 0.006). The same was observed for the country's income status as significantly more departments from HIC (33%; n = 76) than from LMIC (15%; n = 28) indicated owning/using a robot for spinal instrumentations (p < 0.001) (Table 3). Also, the number of annual instrumentation procedures was a significant factor for robotic utilization as departments with high annual rates stated the highest numbers of robotic possessions (38%; n = 43) (p < 0.001). However, no significant differences were observed based on the academic level of the workplace (p = 0.348).

Further assessment revealed that 11% (n = 47) of all participants use a spinal robot frequently (1-2x/week), while 8% (n = 34) use one occasionally (1-2x/month), and 5% (n = 23) possess one but don't use it at all. While 35% (n = 150) of participants planned to acquire a robotic system for spine surgery in the future, a similar number (36%, n = 153) stated they did not want or need one from a current perspective. Altogether, most surgeons (57%, n = 225) indicated a desire to use a robot in the future. Still, most surgeons who use a robot (11%, n = 43) do this only in selected cases, and only 7% (n = 27) use it routinely as standard equipment (Fig. 4).

**Fig. 5:**
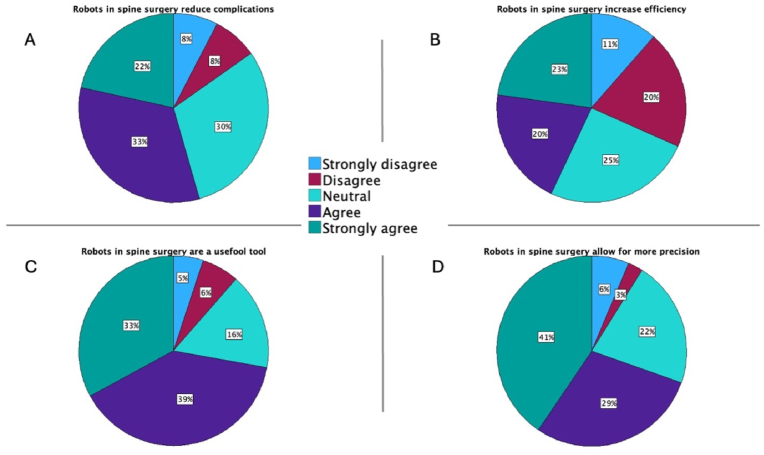
A–D Questions 15–18: Pie charts demonstrating the participants' opinion on the following statements: robots in spine surgery help reduce surgical complications, robots in spine surgery increase intraoperative efficiency, robots in spine surgery are a useful tool, robots in spine surgery allow for more precision during spinal instrumentation/hardware placement.

Nineteen percent (n = 79) of participants identified themselves as robotic users and answered further questions on precision, efficacy, and complications of current systems. The majority of participants (70%, n = 55) agreed (29%, n = 23) or strongly agreed (41%, n = 32) that robots increase precision during spinal instrumentation procedures (Fig. 5A). Approximately half (55%, n = 43) agreed (33%, n = 26) or strongly agreed (22%, n = 17) that robots in spine surgery help reduce surgical complications (Fig. 5B). Fewer participants (43%, n = 34) agreed (20%, n = 16) or strongly agreed (23%, n = 18) regarding the improvement of intraoperative efficiency by spinal robotics, such as the reduction of surgical time or blood loss (Fig. 5C). Three out of four participants (72%, n = 57) believed (39%, n = 31) or strongly believed (33%, n = 26) that robots in spine surgery are useful tools (Fig. 5D).

### 3.5 Future implementation of robotics in spine surgery

Most respondents believed that robotics might positively impact outcomes in MIS (67%, n = 264), adult deformities (62%, n = 244), and pediatric deformities (50%, n = 196). The primary reasons cited for not implementing spinal robot in their department were high acquisition costs (77%, n = 301), followed by insufficient perceived benefits (28%, n = 109), and the perception of sufficient precision with spinal navigation systems alone (20%, n = 79). Participants expected spinal robotic systems to provide high precision with self-control for patient/OR table positioning (63%, n = 248), additional tools for optimizing MIS approaches (62%, n = 243), and reduction of surgical time (54%, n = 212). Participants desired more integrated surgical options (e.g., decompression, discectomy, endoscopy) (70%, n = 271), integration of artificial intelligence with self-updating software platforms (55%, n = 211), and smaller, less bulky devices (53%, n = 206).

We aimed to estimate the current opinion on the efficiency of robotic systems in terms of reduction of revision surgeries, surgical times, and hospital readmissions. Most participants (23%, n = 98) believed that at least 1–5 revision surgeries per year should be avoided, surgical times should be reduced by 31–60 min per day (30%, n = 129), and an average of 6–10 unplanned hospital readmissions per year (18%, n = 77) should be prevented to justify the merit/benefit of robotic surgery. Admittedly, there was no consensus on the exact numbers in these categories and there was a nearly homogeneous distribution of answers.

Finally, we asked the participants what kind of investment they would consider for purchasing a robotic system and when they believe that a spinal robot will become completely autonomous in the future. Most surgeons (43%, n = 166) preferred shared company investment with guaranteed use of implants or products by their hospital, followed by robot leasing (37%, n = 143), and one-time capital investment by the hospital (34%, n = 132). Most participants believed that spinal robotics would become autonomous within 6–10 years (27.2%, n = 116) or never (21.5%, n = 92).

## 4 Discussion

### 4.1 Current use of spinal navigation

The survey results indicate that less than half of participating AO Spine members (42%) use spinal navigation regularly. This finding is consistent with previous studies demonstrating that computer-assisted navigation offers significant benefits in terms of reducing the ionizing radiation exposure (Bratschitsch et al., 2019) and improving pedicle screw accuracy not only in the thoracolumbar spine (Tjardes et al., 2010; Meng et al., 2016), but also in the anatomically more challenging cervical area (Soliman et al., 2023; Lange et al., 2021; Tarawneh et al., 2021), as well as for pediatric and adult deformities (Baldwin Kdk et al., 2022; De Vega et al., 2022). Recent data analysis even suggested that navigated and robotic pedicle screws might even be safer and more accurate than fluoroscopic freehand screws(Patel et al., 2023; Matur et al., 2023; Li et al., 2020). Nevertheless, navigation has not yet become a standard practice on a global scale.

Surprisingly, one-third (33%, n = 142) of participants in this survey declared they had never used a navigation system before, and 13% (n = 55) stated they did not want or need to acquire one at all. Sub-analysis revealed that mainly surgeons with higher caseload (>120 annual instrumentations) working in HIC were more willing to use spinal navigation routinely. This association may be attributed to several factors, including financial considerations, expertise, and workflow efficiency.

Evidence suggests that spinal navigation reduces mispositioned pedicle screws and complications requiring revision surgeries, albeit at the cost of longer surgical times(Meng et al., 2016; Soliman et al., 2023; Tarawneh et al., 2021; De Vega et al., 2022). Surgeons with higher caseloads may benefit from increased expertise and more efficient workflow with intraoperative navigation, potentially shortening image acquisition and reference times. Additionally, while navigation procedures result in higher radiation doses for patients compared to fluoroscopy, spine surgeons can perform up to 10-fold the number of surgeries (10.000 versus 883) with navigation until the maximum permissible annual effective radiation dose is reached (Bratschitsch et al., 2019). This finding again justifies the utilization of spinal navigation by surgeons with a higher annual number of instrumentation procedures. On the other hand, the acquisition of a spinal navigation system might be unaffordable for clinics with a lower annual number of instrumentation procedures for amortization since the costs of the most common robotic systems are estimated in a range between $700.000 and $1.500.000(19), which is almost double the price of a navigation system.

In terms of 3D imaging technique, most respondents (24.5%, n = 104) indicated the use of a cone-beam CT, which offers significant advantages in accuracy over conventional C-arm fluoroscopy(Feng et al., 2020). This preference reflects a balance between flexibility and image quality, contributing to the broader adoption of spinal navigation systems.

### 4.2 Current use of spinal robotics

The survey results reveal that only a limited number of spine surgeons have utilized spinal robotics during real patient procedures, with the majority originating from HIC in Europe, Asia, the Middle East, and North America. This finding underscores the limited accessibility of this technology worldwide, consistent with the geographical distribution of published research on spinal robotics in the USA, Germany, and China(Mualem et al., 2022). Interestingly, only half of the robotic users in our survey stated frequent utilization of their robotic systems, and 5% admitted to possessing a robotic system but not utilizing it at all. This discrepancy may reflect the challenges associated with integrating spinal robotics into clinical practice, including the need for comprehensive workflows and specially trained surgical staff.

While some studies have demonstrated a reduction in surgical times after an initial learning curve with spinal robotics (Pennington et al., 2022), current robotic systems remain cost-intensive (Malham and Wells-Quinn, 2019) and require a comprehensive workflow and specially trained OR/surgical staff (D'Souza et al., 2019). Most robotic systems on the market are still bulky and might lead to prolonged surgical times due to their complex setups (Malham and Wells-Quinn, 2019). Additionally, certain limitations, such as the inability to prevent skiving from the facet joints during pedicle screw insertion(Joseph et al., 2017; Ghasem et al., 2018) and the lack of automatic implant planning, contribute to the ongoing debate regarding the utility of spinal robotics (Ghasem et al., 2018; Marcus et al., 2014; Shweikeh et al., 2014; Keric et al., 2017; Huang et al., 2020; Tarawneh and Salem, 2021). Even though approximately one-third (35%, n = 150) of the respondents indicated they would like to acquire a robotic system at their institution, another third (36%, n = 153) believed they did not need one from a current perspective. Despite the growing popularity of spinal robotic systems in recent years(D'Souza et al., 2019; Stumpo et al., 2021), there is still no consensus on their usefulness. Most robotic users in our survey believe in the precision-enhancing capabilities of robots during instrumentation procedures (69%, n = 55) and that they are useful tools (72%, n = 57). However, opinions regarding the reduction of intraoperative complications and increased surgical efficiency remain inconclusive. This aligns with previous RCT and real-world data demonstrating the high precision of spine robotics (Li et al., 2020; Peng et al., 2024) but similar radiation exposure and complication rates compared to navigated and freehand techniques (Tarawneh et al., 2021; Keric et al., 2017; Huang et al., 2020; Solomiichuk et al., 2017; Staartjes et al., 2018).

### 4.3 Future developments

Survey respondents expressed optimism regarding the potential benefits of robotic assistance in spine surgery, especially in MIS surgeries (67%, n = 264), adult (62%, n = 244), or pediatric (50 %, n = 196) deformities. Previous studies have suggested that robotic assistance in selected cases might facilitate the conversion of open surgery to MIS approaches, ultimately reducing patient length of stay and hospital costs(D'Souza et al., 2019; Menger et al., 2018; Hyun et al., 2017). In deformity cases, robotic assistance might support less experienced surgeons to improve their accuracy and surgical times(Ueno et al., 2023).

However, participants also pointed out significant barriers to the widespread adoption of robotic assistance, including high acquisition costs(Shweikeh et al., 2014) and logistical challenges such as time-consuming setups requiring the simultaneous use of fluoroscopy or other imaging modalities and the need for specialized training for OR staff (Shweikeh et al., 2014). Although robotic assistance was not only applied for accurate percutaneous pedicle screw placement in prone or lateral single position surgery (Patel et al., 2023) in the past but also for comprehensive procedures, e.g., interbody placement in lateral lumbar interbody fusion (LLIF) (Patel et al., 2023; Dalton et al., 2021), laminectomy in models (Li et al., 2022), surgical resection of the spinal column, planning and executing deformity procedures (Haider et al., 2023), augmentative procedures, complex revision surgeries (Overley et al., 2017)^,^ (Haider et al., 2022) and ALIF surgeries (Lee et al., 2013), its current application remains limited primarily to internal spinal fixation (Huang et al., 2020).

Recent studies have reported comparable accuracy between spinal navigation and robotic systems(D'Souza et al., 2019; Patel et al., 2023; Staartjes et al., 2018; Overley et al., 2017), raising questions about the additional benefits of robotic assistance given its higher costs. This suggests that robotic technology may require further clinical and health-economic data to justify its utilization(Malham and Wells-Quinn, 2019). Although robotics in spine surgery offers potential benefits such as increased accuracy and improved outcomes, it also raises concerns about sustainability and environmental impact (D'Souza et al., 2019; Shweikeh et al., 2014). Studies from other surgical fields already demonstrated that robotic procedures result in higher greenhouse gas emissions and waste production compared to established alternatives (Papadopoulou et al., 2022). Robotic-assisted surgery may also prolong the operative times (Ghasem et al., 2018), especially in the early adoption phase. Operating rooms are major contributors to biomedical waste and energy consumption (Talibi et al., 2022), and some of the main strategies to reduce the environmental footprint of spine surgery include minimizing single-use instruments, proper waste segregation, recycling, and adopting carbon-conscious procurement (McNamee et al., 2023; Phoon et al., 2022). Considering the health-economic situation, especially in LMIC with OR staff reduction and the additional costs for disposables, the development of smarter systems with a better carbon footprint and basic workflows might be essential for robotics distribution.

Respondents identified several areas for further development in robotic assistance, including the integration of additional surgical options (e.g., decompression, discectomy, endoscopy), as well as the incorporation of artificial intelligence and the design of smaller, more user-friendly devices. More attractive financial solutions, such as shared company investments with guaranteed use of implants or other products by the hospital, leasing options, or one-time capital investment by the hospital, may also contribute to increased distribution and utilization of robotic assistance in spine surgery.

Ultimately the timeline for the complete autonomy of robots remains uncertain, with respondents indicating varying perspectives on the feasibility of this development within 6–10 years. Continued research and technological advancements will be essential to address current limitations and maximize the potential of robotic assistance in spine surgery.

### 4.4 Strengths and limitations

In this study, we provide valuable insights into the current state of navigation and robotics in spine surgery. Our ability to gather responses from a diverse group of participants, primarily AO Spine members, enhances the generalizability of our findings and contributes to a more representative understanding of current practices. The inclusion of participants from different professional and geographical backgrounds enriches the breadth and depth of our analysis, allowing for a more nuanced exploration of trends and preferences in spine surgery technologies.

However, it is important to acknowledge the limitations inherent to our study methodology. As with all survey-based research, our data collection relied on voluntary, non-incentivized participation, which may introduce selection bias. Participants who chose to respond to the survey may have distinct perspectives or experiences compared to those who did not participate, potentially influencing the responsiveness of our sample. Additionally, the subjective nature of self-reporting may introduce response bias, further limiting the validity of our data.

While we achieved a reasonably high response rate, it is essential to recognize that our findings may not fully capture the complexity of healthcare settings or the overall adoption of robotics in spine surgery. The data presented in our study should be interpreted with caution, considering the inherent limitations of survey-based research. Despite these limitations, our study provides valuable insights into current practices and highlights areas for further investigation and improvement in the implementation of navigation and robotics in spine surgery.

## 5 Conclusions

Robotics in spine surgery currently exhibits lower popularity among spine surgeons compared to spinal navigation, as evidenced by our study findings. While some institutions in HIC across Europe, Asia, the Middle East, and North America with a high volume of instrumentation procedures have embraced robotic assistance, its widespread adoption remains limited. Despite the perceived benefits of robotics in MIS and deformity surgery, challenges such as high acquisition costs and limited demonstrated benefits hinder its current implementation in daily practice. It is imperative for the field to prioritize the advancement of strategies to overcome barriers to implementation, alongside the accumulation of robust scientific evidence to support the standardized utilization of robotics in spine surgery.

## Declaration of competing interest

The authors declare that they have no known competing financial interests or personal relationships that could have appeared to influence the work reported in this paper.

## References

[bib1] Baldwin Kdk M., Talwar D., Sankar W.N., Flynn J.J.M., Anari J.B. (2022). Does intraoperative CT navigation increase the accuracy of pedicle screw placement in pediatric spinal deformity surgery? A systematic review and meta‐analysis. Spine Deformity..

[bib2] Bratschitsch G., Leitner L., Stucklschweiger G., Guss H., Sadoghi P., Puchwein P. (2019). Radiation exposure of patient and operating room personnel by fluoroscopy and navigation during spinal surgery. Sci. Rep..

[bib3] D'Souza M., Gendreau J., Feng A., Kim L.H., Ho A.L., Veeravagu A. (2019). Robotic-assisted spine surgery: history, efficacy, cost, and future trends. Robot Surg.

[bib4] Dalton T., Sykes D., Wang T.Y., Donnelly D., Than K.D., Karikari I.O. (2021). Robotic-assisted trajectory into kambin's triangle during percutaneous transforaminal lumbar interbody fusion-initial case series investigating safety and efficacy. Oper Neurosurg (Hagerstown)..

[bib5] De Vega B., Navarro A.R., Gibson A., Kalaskar D.M. (2022). Accuracy of pedicle screw placement methods in pediatrics and adolescents spinal surgery: a systematic review and meta-analysis. Global Spine J..

[bib6] Feng W., Wang W., Chen S., Wu K., Wang H. (2020). O-arm navigation versus C-arm guidance for pedicle screw placement in spine surgery: a systematic review and meta-analysis. Int. Orthop..

[bib7] Ghasem A., Sharma A., Greif D.N., Alam M., Maaieh M.A. (2018). The arrival of robotics in spine surgery: a review of the literature. Spine.

[bib8] Haider G., Wagner K.E., Chandra V., Cheng I., Stienen M.N., Veeravagu A. (2022). Utilization of lateral anterior lumbar interbody fusion for revision of failed prior TLIF: illustrative case. J Neurosurg Case Lessons.

[bib9] Haider G., Shah V., Johnstone T., Maldaner N., Stienen M., Veeravagu A. (2023). Accuracy of predicted postoperative segmental lumbar lordosis in spinal fusion using an intraoperative robotic planning and guidance system. J. Neurosurg. Sci..

[bib10] Huang J., Li Y., Huang L. (2020). Spine surgical robotics: review of the current application and disadvantages for future perspectives. J Robot Surg.

[bib11] Hyun S.J., Kim K.J., Jahng T.A., Kim H.J. (2017). Minimally invasive robotic versus open fluoroscopic-guided spinal instrumented fusions: a randomized controlled trial. Spine.

[bib12] Joseph J.R., Smith B.W., Liu X., Park P. (2017). Current applications of robotics in spine surgery: a systematic review of the literature. Neurosurg. Focus.

[bib13] Keric N., Doenitz C., Haj A., Rachwal-Czyzewicz I., Renovanz M., Wesp D.M.A. (2017). Evaluation of robot-guided minimally invasive implantation of 2067 pedicle screws. Neurosurg. Focus.

[bib14] Kochanski R.B., Lombardi J.M., Laratta J.L., Lehman R.A., O'Toole J.E. (2019). Image-guided navigation and robotics in spine surgery. Neurosurgery.

[bib15] Lange N., Meyer B., Meyer H.S. (2021). Navigation for surgical treatment of disorders of the cervical spine - a systematic review. J. Orthop. Surg..

[bib16] Lee J.Y., Bhowmick D.A., Eun D.D., Welch W.C. (2013). Minimally invasive, robot-assisted, anterior lumbar interbody fusion: a technical note. J. Neurol. Surg. Cent. Eur. Neurosurg..

[bib17] Li H.M., Zhang R.J., Shen C.L. (2020). Accuracy of pedicle screw placement and clinical outcomes of robot-assisted technique versus conventional freehand technique in spine surgery from nine randomized controlled trials: a meta-analysis. Spine.

[bib18] Li Z., Jiang S., Song X., Liu S., Wang C., Hu L. (2022). Collaborative spinal robot system for laminectomy: a preliminary study. Neurosurg. Focus.

[bib19] Liounakos J.I., Chenin L., Theodore N., Wang M.Y. (2021). Robotics in spine surgery and spine surgery training. Oper Neurosurg (Hagerstown).

[bib20] Malham G.M., Wells-Quinn T. (2019). What should my hospital buy next?-Guidelines for the acquisition and application of imaging, navigation, and robotics for spine surgery. J Spine Surg.

[bib21] Marcus H.J., Cundy T.P., Nandi D., Yang G.Z., Darzi A. (2014). Robot-assisted and fluoroscopy-guided pedicle screw placement: a systematic review. Eur. Spine J..

[bib22] Matur A.V., Palmisciano P., Duah H.O., Chilakapati S.S., Cheng J.S., Adogwa O. (2023). Robotic and navigated pedicle screws are safer and more accurate than fluoroscopic freehand screws: a systematic review and meta-analysis. Spine J..

[bib23] McNamee C., Rakovac A., Cawley D.T. (2023). The environmental impact of spine surgery and the path to sustainability. Spine.

[bib24] Meng X.T., Guan X.F., Zhang H.L., He S.S. (2016). Computer navigation versus fluoroscopy-guided navigation for thoracic pedicle screw placement: a meta-analysis. Neurosurg. Rev..

[bib25] Menger R.P., Savardekar A.R., Farokhi F., Sin A. (2018). A cost-effectiveness analysis of the integration of robotic spine technology in spine surgery. Neurospine.

[bib26] Mualem W., Onyedimma C., Ghaith A.K., Durrani S., Jarrah R., Singh R. (2022). R2 advances in robotic-assisted spine surgery: comparative analysis of options, future directions, and bibliometric analysis of the literature. Neurosurg. Rev..

[bib27] Overley S.C., Cho S.K., Mehta A.I., Arnold P.M. (2017). Navigation and robotics in spinal surgery: where are we now?. Neurosurgery.

[bib28] Papadopoulou A., Kumar N.S., Vanhoestenberghe A., Francis N.K. (2022). Environmental sustainability in robotic and laparoscopic surgery: systematic review. Br. J. Surg..

[bib29] Patel N.A., Kuo C.C., Pennington Z., Brown N.J., Gendreau J., Singh R. (2023). Robot-assisted percutaneous pedicle screw placement accuracy compared with alternative guidance in lateral single-position surgery: a systematic review and meta-analysis. J. Neurosurg. Spine.

[bib30] Peng J., Li Q., Zhang X., Li J., Wan S., Yu S. (2024). Safety and accuracy of robot-assisted cervical screw placement: a systematic review and meta-analysis. World Neurosurg.

[bib31] Pennington Z., Judy B.F., Zakaria H.M., Lakomkin N., Mikula A.L., Elder B.D. (2022). Learning curves in robot-assisted spine surgery: a systematic review and proposal of application to residency curricula. Neurosurg. Focus.

[bib32] Phoon K.M., Afzal I., Sochart D.H., Asopa V., Gikas P., Kader D. (2022). Environmental sustainability in orthopaedic surgery : a scoping review. Bone Jt Open.

[bib33] Shweikeh F., Amadio J.P., Arnell M., Barnard Z.R., Kim T.T., Johnson J.P. (2014). Robotics and the spine: a review of current and ongoing applications. Neurosurg. Focus.

[bib34] Soliman M.A.R., Aguirre A.O., Khan S., Kuo C.C., Ruggiero N., Mariotti B.L. (2023). Complications associated with subaxial placement of pedicle screws versus lateral mass screws in the cervical spine (C2-T1): systematic review and meta-analysis comprising 4,165 patients and 16,669 screws. Neurosurg. Rev..

[bib35] Solomiichuk V., Fleischhammer J., Molliqaj G., Warda J., Alaid A., von Eckardstein K. (2017). Robotic versus fluoroscopy-guided pedicle screw insertion for metastatic spinal disease: a matched-cohort comparison. Neurosurg. Focus.

[bib36] Staartjes V.E., Klukowska A.M., Schroder M.L. (2018). Pedicle screw revision in robot-guided, navigated, and freehand thoracolumbar instrumentation: a systematic review and meta-analysis. World Neurosurg.

[bib37] Stumpo V., Staartjes V.E., Klukowska A.M., Golahmadi A.K., Gadjradj P.S., Schroder M.L. (2021). Global adoption of robotic technology into neurosurgical practice and research. Neurosurg. Rev..

[bib38] Talibi S.S., Scott T., Hussain R.A. (2022). The environmental footprint of neurosurgery operations: an assessment of waste streams and the carbon footprint. Int J Environ Res Public Health.

[bib39] Tarawneh A.M., Salem K.M. (2021). A systematic review and meta-analysis of randomized controlled trials comparing the accuracy and clinical outcome of pedicle screw placement using robot-assisted technology and conventional freehand technique. Global Spine J..

[bib40] Tarawneh A.M., Haleem S., D'Aquino D., Quraishi N. (2021). The comparative accuracy and safety of fluoroscopic and navigation-based techniques in cervical pedicle screw fixation: systematic review and meta-analysis. J. Neurosurg. Spine.

[bib41] Tjardes T., Shafizadeh S., Rixen D., Paffrath T., Bouillon B., Steinhausen E.S. (2010). Image-guided spine surgery: state of the art and future directions. Eur. Spine J..

[bib42] Ueno J., Torii Y., Umehra T., Iinuma M., Yoshida A., Tomochika K. (2023). Robotics is useful for less-experienced surgeons in spinal deformity surgery. Eur. J. Orthop. Surg. Traumatol..

