# Supplementary material for: Current state and future perspectives of spinal navigation and robotics—an AO spine survey
Source: Brain Spine. 2024 Dec 18;5:104165. doi: 10.1016/j.bas.2024.104165 (PMC11732222; doi:10.1016/j.bas.2024.104165)
Supplement: Multimedia component 1 [file mmc1.docx]

**Appendix 1:**

**Survey on spinal robotics**

*Please answer the following questions if you have any experience or already utilize spinal navigation and/or robotics in your practice. Surgeons who are not experienced with these technologies are also kindly asked to participate in this survey, but we request to state your level of knowledge and experience.*

1. **Age**

_____________

1. **Gender (single answer)**
2. Female
3. Male
4. Other
5. **Training/background (single answer)**
6. Board-certified in neurosurgery
7. Board-certified in orthopedics
8. Board-certified in trauma surgery
9. Resident in neurosurgery
10. Resident in orthopedics
11. Resident in trauma surgery
12. Other (please specify): ______________
13. **In which country do you practice? (single answer)**

____________

1. **Workplace (single answer)**
2. University hospital
3. Public, non-university hospital
4. Private hospital/practice
5. Other (please specify): ____________
6. **How many spinal instrumentation/fusion procedures do you personally perform/year? (single answer)**
7. <30
8. 30-60
9. 60-120
10. 120-250
11. >250
12. None
13. **Please state which technique do you employ in your practice to insert pedicle screws: (multiple answers)**
14. Free hand with X-ray confirmation
15. Free hand with fluoroscopy
16. Fluoro guidance
17. Navigation assisted
18. Robotic assisted
19. **Have you ever used intraoperative 3D-navigation during a real patient procedure for spinal instrumentation (excluding cadaver lab, training course, industry booth, etc.)? (single answer)**
20. No
21. Yes, during residency/fellowship training at another department
22. Yes, at our department
23. **Does your department own/use a navigation system for spinal instrumentation? (single answer)**
24. Yes, and we use it frequently (1-2 times a week)
25. Yes, but we use it only occasionally (1-2 times/a month)
26. Yes, but we don’t use it at all currently
27. No, but we are planning to acquire one
28. No, and we don’t want/need it from a current perspective
29. Does the navigation system used at your hospital include intraoperative 3D imaging (intraoperative CT, conebeam-CT, 3D C-arm)?
30. Yes, it includes a CT (e.g., Airo CT)
31. Yes, it includes a conebeam-CT (e.g., O-Arm)
32. Yes, it includes a 3D C-Arm
33. Yes, other: ________
34. No, but we are planning to acquire one
35. No, and we don’t want/need it from a current perspective
36. **Do you personally use a navigation system for spinal instrumentation in your surgical routine? (single answer)**
37. Yes, routinely (standard for spinal instrumentation)
38. Yes, but only in selected cases (not for standard cases)
39. No, but I would like to
40. No, and I do not want to
41. Other: (please specify) ______
42. **Have you ever used a robot (e.g., *Cirq, MazorX, Excelsius-GPS, ROSA* etc.) during a real patient procedure for spinal instrumentation (excluding cadaver lab, training course, industry booth, etc.)? (single answer)**
43. No
44. Yes, during residency/fellowship training at another department (please specify which system):
    1. Brainlab Cirq
    2. Medtronic Mazor X
    3. Globus Excelsius-GPS
    4. Zimmer Biomet ROSA
    5. Other (please specify): ________
45. Yes, at our department (please specify which system):
    1. Brainlab Cirq
    2. Medtronic Mazor X
    3. Globus Excelsius-GPS
    4. Zimmer Biomet ROSA
    5. Other (please specify): ________
46. **Does your department own/use a robot for spinal instrumentation? (single answer)**
47. Yes, and we use it frequently (1-2 times a week)
48. Yes, but we use it only occasionally (1-2 times/a month)
49. Yes, but we don’t use it at all currently
50. No, but we are planning to acquire one
51. No, and we don’t want/need it from a current perspective
52. **Do you personally use a robot for spinal instrumentation in your surgical routine? (single answer)**
53. Yes, routinely (standard for spinal instrumentation)
54. Yes, but only in selected cases (not for standard cases)
55. No, but I would like to
56. No, and I do not want to
57. Other: (please specify) ______

*The following questions 13)-16) should only be answered by those who stated to use a robotic system occasionally (1-2 times/a month) or frequently (1-2 times a week).*

**Please provide your current perspective/opinion on the following statements:**

1. **Robots in spine surgery allow for more precision during spinal instrumentation / hardware placement (single answer).**
2. Strongly agree
3. Agree
4. Neutral
5. Disagree
6. Strongly disagree
7. **Robots in spine surgery help reduce surgical complications (single answer).**
8. Strongly agree
9. Agree
10. Neutral
11. Disagree
12. Strongly disagree
13. **Robots in spine surgery increase intraoperative efficiency (which may help shorten OR times and reduce blood loss) (single answer).**
14. Strongly agree
15. Agree
16. Neutral
17. Disagree
18. Strongly disagree
19. **Robots in spine surgery are a useful tool (single answer).**
20. Strongly agree
21. Agree
22. Neutral
23. Disagree
24. Strongly disagree
25. **What pathology do you think robotics can improve care on or make impact on outcome (multiple answers possible):**
26. Degenerative
27. Trauma
28. Infection
29. Tumor
30. Adult deformity
31. Pediatric deformity
32. MIS (minimally-invasive spine surgery)
33. I don’t use spinal robotics
34. **If you haven’t implemented a spinal robot in your department yet, why not**

**(multiple answers possible)?**

1. High acquisition costs
2. I don’t think that a robot has enough benefit yet (only use as “drill guide” so far)
3. The precision of navigation only is high enough, no need for additional robotics
4. The implementation and use are too time-consuming with spinal robotics
5. The workflow is too complicated with spinal robotics
6. The available systems are too bulky
7. I want to train my residents in the “free-hand technique” for spinal instrumentation and the robot would interfere with training
8. **What are your expectations to a robotic system currently (multiple answers possible)?**
9. Reducing surgical times
10. Tools for creation and optimization of minimally-invasive approaches
11. (Semi-)Automatic planning of implants
12. High precision with self-control for patient/OR table positioning
13. Automatic drilling & implant insertion
14. Remote control for remote use
15. Other (please specify): _______________
16. **What future innovations do you wish for spinal robotics (multiple answers possible)?**
17. Smaller devices
18. Higher robotic autonomy (automatic screw placement)
19. More surgical options integrated (e.g., decompression, discectomy, endoscopy) – not “only drill guide”
20. Integration of artificial intelligence with self-updating software platform
21. Remote control with mixed reality tools (e.g., remote control with hand-eye tracking)
22. Simultaneous robotic activity (bilateral screw placement, decompression and irrigation)
23. Other (please specify):___________________
24. **In your opinion, how many revision surgeries per year must be avoided by use of a spinal robot in order to have a clear merit / benefit (single answer)?**
25. 1-5
26. 5-10
27. 10-20
28. 20-30
29. > 30
30. None of the above.
31. **In your opinion, how much surgical time should be saved per day through the implementation of a spinal robot in order to have a clear merit / benefit (single answer)?**
32. <15 minutes
33. 15-30 minutes
34. 30-60 minutes
35. >60 minutes
36. None of the above.
37. **In your opinion, how many unplanned hospital re-admissions should be prevented per year through robotic surgery in order to have a clear merit / benefit (single answer)?**
38. 1-3
39. 3-5
40. 5-10
41. 10-20
42. >20
43. None of the above.
44. **If you wish to purchase a spine robot, which of the following options would you consider (multiple answers)?**
45. One-time capital investment (all costs paid by the hospital)
46. Leasing (annual rates paid by the hospital)
47. Shared company investment with guaranteed use of implants or other products by the hospital
48. Shared hospital and state funding
49. None of the above

1. **When do you believe that a spinal robot will be able to autonomously complete a spinal surgery (single answer)?**
2. 1-5 years from now
3. 5-10 years from now
4. 10-20 years from now
5. >20 years from now
6. Never
